# Genotyping and distribution of putative virulence factors and antibiotic resistance genes of *Acinetobacter baumannii* strains isolated from raw meat

**DOI:** 10.1186/s13756-018-0405-2

**Published:** 2018-10-04

**Authors:** Marziyeh Tavakol, Hassan Momtaz, Parviz Mohajeri, Leili Shokoohizadeh, Elahe Tajbakhsh

**Affiliations:** 1 Department of Microbiology, Shahrekord Branch, Islamic Azad University, PO. Box: 166, Shahrekord, Iran; 2 Department of Microbiology, School of Medicine, Kermanshah University of Medical Sciences, Kermanshah, Iran; 3 Nosocomial Infection Research Center, Kermanshah University of Medical Sciences, Kermanshah, Iran; 4 Department of Microbiology, School of Medicine, Hamadan University of Medical Sciences, Hamadan, Iran

**Keywords:** *Acinetobacter baumannii*, ERIC-PCR, Virulence genes, Antibiotic resistance pattern, Raw meat

## Abstract

**Background:**

*Acinetobacter baumannii* strains with multiple antimicrobial resistance are primarily known as opportunistic nosocomial bacteria but they may also be regarded as emerging bacterial contaminants of food samples of animal origin. Here we aimed to study the molecular characteristics of the *A. baumanni* strains isolated from raw meat samples.

**Methods:**

A total of 22 *A. baumanni* strains were isolated from 126 animal meat samples and were genotyped by ERIC-PCR method and by PCR detection of their virulence and antimicrobial resistance determinants. *A. baumannii* strains with 80% and more similarities were considered as one cluster.

**Results:**

Sixteen different genetic clusters were found amongst the 22 *A. baumanni* strains. Of the 22 strains, 12 (54.54%) had similar genetic cluster. *A. baumannii* strains exhibited the highest percentage of resistance against tetracycline (90.90%), trimethoprim (59.09%), cotrimoxazole (54.54%) and gentamicin (50.00%). *TetA* (81.81%), *tetB* (72.72%), *dfrA1* (63.63%), *aac(3)-IV* (63.63%), *sul1* (63.63%) and *aadA1* (45.45%) were the most commonly detected antibiotic resistance genes. *FimH* (81.81%), *afa/draBC* (63.63%), *csgA* (63.63%), *cnf1* (59.09%), *cnf2* (54.54%) and *iutA* (50.00%) were the most commonly detected virulence factors. *A. baumannii* strains isolated from the chicken meat samples had the highest similarities in the genetic cluster.

**Conclusions:**

*A. baumannii* strains with similar genetic cluster (ERIC-Type) had the same prevalence of antibiotic resistance, antibiotic resistance genes and virulence factors. Genetic cluster of the *A. baumannii* strains is the main factor affected the similarities in the genotypic and phenotypic properties of the *A. baumannii* strains.

## Background

Consumption of raw or undercooked meat has been associated with several outbreaks of bacterial foodborne disease all-around the world [1–5]. Bacterial strains isolated from meat are both pathogenic and commensal such as *Staphylococcus aureus*, *Streptococcus* species, *Listeria monocytogenes*, Bacillus spp., Salmonella spp., Campylobacter spp., *Escherichia coli*, *Yersinia enterocolitica*, Acinetobacter spp., Aeromonas spp., Pseudomonas spp. and etc. [2, 3, 6–9]. *Acinetobacter* species are saprophytic, ubiquitous and have emerged as an important nosocomial pathogen due to its ability for survival in the hospital environment on a wide range of dry and moist surfaces [10, 11]. Human infections caused by Acinetobacter species include pneumonia, which is most often related to endotracheal tubes or tracheostomies, endocarditis, meningitis, skin and wound infections, peritonitis in patients receiving peritoneal dialysis, UTI and bacteremia.[10, 11] Acinetobacter spp. and especially *Acinetobacter baumannii* are one of the newly emerged bacteria all-around the world [10, 11]. *A. baumannii* colonizes the respiratory tract, skin, urinary and gastrointestinal systems, and frequently leads to pneumonia, surgical site infections, catheter-related blood circulatory infections, and urinary tract infections and rarely community acquired pneumonia, meningitis, mediastinitis, osteomyelitis and cholangitis [10, 11].

*A. baumannii* strains isolated from clinical infections harbored the high prevalence of certain virulence factors including pathogenicity islands (PAI), P fimbriae (*pap* genes), S fimbriae adhesion and F1C (*sfa*), a fimbriae adhesion and Dr. antigen family (*afa*/*dra*), type 1 fimbriae (*fimH*), fibronectin receptor (*fbn*), serum resistance (*traT*), curli fibers (*csg*), aerobactin (*iutA*), yersiniabactin (*fyuA*), polysaccharide coatings as group II and III capsules (*kpsMT*), cytotoxic necrotizing factors (*cnf*), invasins factors (*IbeA*) and colicin V production (*cvaC*). Moreover, these factors are responsible for adhesion, colonization and invasion of the *A. baumannii* strains into the different target organs [12, 13].

*A. baumannii* strains are also known to harbor multiple resistance against different types of antibiotics. Documented data revealed that the *A. baumannii* strains isolated from human and animal clinical infections and food stuffs harbored the high prevalence of resistance against different types of antibiotics [14, 15]. The genes that encode resistance against antibiotics and especially *aadA1* (streptomycin), *aac(3)-IV* (gentamicin), *sul1* (sulfonamide), *blaSHV* and *blaCTX-M* (cephalosporins), *cat1* and *cmlA* (chloramphenicol), *tetA* and *tetB* (tetracycline), *dfrA1* (trimethoprim), *qnr* (fluoroquinolones)*,imp*, *sim* and *vim* (carbenicillin), and *Oxa-51-like*(oxacillinases**)** are mainly responsible for occurrence of severe antibiotic resistance [14–16]. In keeping with this, genotyping is one of the best techniques to find the molecular relationship between bacterial strains isolated from different sources. Enterobacterial Repetitive Intergenic Consensus Polymerase Chain Reaction (ERIC-PCR) is one of the best typing methods of DNA fingerprints of bacterial strains. This method has sporadically been used to characterize *A. baumannii* strains isolated from clinical samples [17].

Studies regarding the association of *Acinetobacter* with foodborne illnesses are somewhat limited [10, 11].Therefore, the present investigation was done to study the phenotypic and genotypic characterization of antibiotic resistance and molecular typing of the *A. baumannii* strains isolated from raw meat samples.

## Methods

### *A. baumannii* strains

From January 2016 to May 2017, a total of 22 *A. baumannii* strains were isolated from 126 samples of raw chicken, turkey, bovine, ovine and camel meat samples. Meat samples were randomly collected (simple random sampling procedure) from the butchers and shopping center of different parts of the Chaharmahal Va Bakhtiari province, Iran. All isolates were transferred to the Microbiology Research Center of the Islamic Azad University of Shahrekord in cooler with ice-packs.

*A. baumanni* strains were isolated based on the method described by the Cappuccino and Welsh (2016) [18]. Briefly, the specimens were inoculated initially on blood agar (Merck) and MacConkey agar (Merck) medium and incubated for 24 h at 37 °C. Conventional biochemical methods such as oxidase, citrate, urea urease, malonate consumption, oxidation and fermentation of sugars, motility and indole production were used to identify *A. baumannii*. Additionally, genus Acinetobacter was identified by Gram staining, cell and colony morphology, positive catalase test, negative oxidase test and absence of motility. Speciation of *Acinetobacter* was performed on the basis of glucose oxidation, gelatin liquefaction, beta hemolysis, growth at 37 °C and 42 °C, arginine hydrolysis and susceptibility to chloramphenicol [18]. The isolates were stored in BHI medium containing 15% glycerol at − 20 °C.

### PCR confirmation of the *A. baumannii* isolates

Confirmation of *A. baumannii* isolates were done using the PCR method. Presence of the16S–23S ribosomal RNA (*16S–23SrRNA*) gene was analyzed using the PCR method described previously [19]. *A. baumannii* isolates were sub-cultured on TSB media (Merck, Germany) and incubated for 48 h at 37 °C. Genomic DNA was extracted from bacterial colonies using the DNA extraction kit (Cinnagen, Iran) according to manufacturer’s instruction. A pair of primers of P-Ab-ITSF (5’-CATTATCACGGTAATTAGTG-3′) and PAb-ITSB (5’-AGAGCACTGTGCACTTAAG-3′) (208 bp) was used for PCR amplification of the *16S–23SrDNA* gene of the *A. baumanni* according to the method described by Chiang et al., (2011) [19]. Confirmed strains were maintained in slant nutrient agar (Merck, Germany) and stored at − 70 °C on 20% sterile buffered glycerol.

### Antibiotic susceptibility testing

Patterns of antimicrobial resistance of the *A. baumanni* strains were studied using the simple disk diffusion technique. The Mueller–Hinton agar (Merck, Germany) medium was used for this purpose. Susceptibility of *A. baumanni*isolates were tested against several types of antibiotics with appropriate disks containing azithromycin (15 μg), erythromycin (15 μg), rifampin (5 μg), nitrofurantoin (300 μg), chloramphenicol (30 μg), imipenem (30 μg), meropenem (10 μg), levofloxacin (5 μg), ciprofloxacin (5 μg), trimethoprim (5 μg), tetracycline (30 μg), ceftazidime (30 μg), cephalotin (30 μg), trimethoprim/sulphamethoxazole(25 μg), tobramycin (10 μg), amikacin (30 μg), gentamicin (10 μg) and streptomycin (10 μg) (produced by PadTan-Teb, Iran), according to the instruction of Clinical and Laboratory Standards Institute [20]. *A. baumanni* ATCC 19606 was used for quality control organism in antimicrobial susceptibility determination.

### Detection of antibiotic resistance genes and virulence factors

Thermo-cycler (Eppendorf Mastercycler 5330, Eppendorf-Nethel-Hinz GmbH, Hamburg, Germany) was used in all PCR reactions. Table 1 represents the list of primers and PCR conditions used for amplification of virulence factors and antibiotic resistance genes in the *A. baumannii* strains.

**Table 1:** Oligonucleotide primers and PCR conditions used for amplification of the antibiotic resistance genes and virulence factors in the A. baumannii strains of animal origin [19, 34–46]

Target gene	Primer Sequence (5′-3′)	Size of product (bp)	PCR conditions	Volume (50 μl)
*draBC*	GCTGGGCAGCAAACTGATAACTCTC CATCAAGCTGTTTGTTCGTCCGCCG	750	1 cycle: 95 0C ------------ 4 min. 30 cycle: 95 0C ------------ 50 s 58 0C ------------ 60 s 72 0C ------------ 45 s 1 cycle: 72 0C ------------ 8 min	5 μL PCR buffer 10X 1.5 mM Mgcl2 200 μM dNTP (Fermentas) 0.5 μM of each primers F & R 1.25 U Taq DNA polymerase (Fermentas) 2.5 μL DNA template
*cnf1*	AAGATGGAGTTTCCTATGCAGGAG CATTCAGAGTCCTGCCCTCATTATT	498		
*csgA*	ACTCTGACTTGACTATTACC AGATGCAGTCTGGTCAAC	200		
*cvaC*	CACACACAAACGGGAGCTGTT CTTCCCGCAGCATAGTTCCAT	680		
*iutA*	GGCTGGACATCATGGGAACTGG CGTCGGGAACGGGTAGAATCG	300		
*fyuA*	TGATTAACCCCGCGACGGGAA CGCAGTAGGCACGATGTTGTA	880		
*cnf2*	AATCTAATTAAAGAGAAC CATGCTTTGTATATCTA	543	1 cycle: 94 0C ------------ 6 min. 34 cycle: 95 0C ------------ 50 s 58 0C ------------ 70 s 72 0C ------------ 55 s 1 cycle: 72 0C ------------ 10 min	5 μL PCR buffer 10X 2 mM Mgcl2 150 μM dNTP (Fermentas) 0.75 μM of each primers F & R 1.5 U Taq DNA polymerase (Fermentas) 3 μL DNA template
*kpsMT* II	GCGCATTTGCTGATACTGTTG CATCCAGACGATAAGCATGAGCA	272		
*PAI*	GGACATCCTGTTACAGCGCGCA TCGCCACCAATCACAGCCGAAC	930		
*papC*	GACGGCTGTACTGCAGGGTGTGGCG ATATCCTTTCTGCAGGGATGCAATA	328		
*fimH*	TGCAGAACGGATAAGCCGTGG GCAGTCACCTGCCCTCCGGTA	508	1 cycle: 95 0C ------------ 4 min. 34 cycle: 94 0C ------------ 60 s 56 0C ------------ 45 s 72 0C ------------ 60 s 1 cycle: 72 0C ------------ 10 min	5 μL PCR buffer 10X 2 mM Mgcl2 200 μM dNTP (Fermentas) 0.5 μM of each primers F & R 1.5 U Taq DNA polymerase (Fermentas) 5 μL DNA template
*ibeA*	AGGCAGGTGTGCGCCGCGTAC TGGTGCTCCGGCAAACCATGC	170		
*PapG* II-III	CTGTAATTACGGAAGTGATTTCTG ACTATCCGGCTCCGGATAAACCAT	1070		
s*fa/focDE*	CTCCGGAGAACTGGGTGCATCTTAC CGGAGGAGTAATTACAAACCTGGCA	410		
*traT*	GGTGTGGTGCGATGAGCACAG CACGGTTCAGCCATCCCTGAG	290		
*aadA1*	TATCCAGCTAAGCGCGAACT ATTTGCCGACTACCTTGGTC	447	1 cycle: 94 0C ------------ 6 min. 33 cycle: 95 0C ------------ 70 s 55 0C ------------ 65 s 72 0C ------------ 90 s 1 cycle: 72 0C ------------ 8 min	5 μL PCR buffer 10X 2 mM Mgcl2 150 μM dNTP (Fermentas) 0. 5 μM of each primers F & R 1.5 U Taq DNA polymerase (Fermentas) 2 μL DNA template
*aac(3)-IV*	CTTCAGGATGGCAAGTTGGT TCATCTCGTTCTCCGCTCAT	286		
*sul1*	TTCGGCATTCTGAATCTCAC ATGATCTAACCCTCGGTCTC	822		
*blaSHV*	TCGCCTGTGTATTATCTCCC CGCAGATAAATCACCACAATG	768		
*CITM*	TGGCCAGAACTGACAGGCAAA TTTCTCCTGAACGTGGCTGGC	462		
*cat1*	AGTTGCTCAATGTACCTATAACC TTGTAATTCATTAAGCATTCTGCC	547		
*cmlA*	CCGCCACGGTGTTGTTGTTATC CACCTTGCCTGCCCATCATTAG	698		
*tet(A)*	GGTTCACTCGAACGACGTCA CTGTCCGACAAGTTGCATGA	577		
*tet(B)*	CCTCAGCTTCTCAACGCGTG GCACCTTGCTGATGACTCTT	634		
*dfrA1*	GGAGTGCCAAAGGTGAACAGC GAGGCGAAGTCTTGGGTAAAAAC	367		
*qnr*	GGGTATGGATATTATTGATAAAG CTAATCCGGCAGCACTATTTA	670		
*imp*	GAATAGAATGGTTAACTCTC CCAAACCACTAGGTTATC	188	1 cycle: 95 0C ------------ 4 min. 30 cycle: 95 0C ------------ 45 s 58 0C ------------ 60s 72 0C ------------ 40 s 1 cycle: 72 0C ------------ 5 min	5 μL PCR buffer 10X 1.5 mM Mgcl2 100 μM dNTP (Fermentas) 1 μM of each primers F & R 1 U Taq DNA polymerase (Fermentas) 2.5 μL DNA template
*vim*	GTTTGGTCGCATATCGCAAC AATGCGCAGCACCAGGATAG	382		
*sim*	GTACAAGGGATTCGGCATCG GTACAAGGGATTCGGCATCG	569		
*Oxa-*23-*like*	GATCGGATTGGAGAACCAGA ATTTCTGACCGCATTTCCAT	501	1 cycle: 94 0C ------------ 5 min. 32 cycle: 95 0C ------------ 50 s 60 0C ------------ 60 s 72 0C ------------ 70 s 1 cycle: 72 0C ------------ 10 min	5 μL PCR buffer 10X 2.5 mM Mgcl2 200 μM dNTP (Fermentas) 0.5 μM of each primers F & R 1.5 U Taq DNA polymerase (Fermentas) 2 μL DNA template
*Oxa-*24-*like*	GGTTAGTTGGCCCCCTTAAA AGTTGAGCGAAAAGGGGATT	246		
*Oxa-51-like*	TAATGCTTTGATCGGCCTTG TGGATTGCACTTCATCTTGG	353		
*Oxa-58-like*	AAGTATTGGGGCTTGTGCTG CCCCTCTGCGCTCTACATAC	599		

### Molecular typing of *A. baumannii* isolates using the ERIC-PCR

The primer pair ERIC1: 5′-ATGTAAGCTCCTGGGGATTCAC-3′ and ERIC2: 5′- AAGTAAGTGACTGGGGTGAGCG-3′ were used to amplify intervening fragments of ERIC in the genomic DNA extracted from the bacterial colonies [17]. Amplification reactions were performed in a final volume of 25 μL including 150 μM dNTP mixture, 1.5 U Taq enzyme, 0.5 μM each primer and 1 μL DNA template. The PCR conditions were as follows: initial denaturation (5 min at 94 °C), followed by four cycles of denaturation (1 min at 94 °C), annealing (1 min at 26 °C) and extension (1 min at 72 °C) and 40 cycles of denaturation (30 s at 94 °C), annealing (30 s at 40 °C) and extension (1 min at 72 °C), followed by the final extension at 72 °C for 10 min [17]. Amplified products of each sample were subjected to electrophoresis in 2% agarose gel containing DNA Safe Stain (CinnaGen, Iran).

### Data analysis

Electrophoretic patterns were analyzed either visually or by using the Bionumerics software (Applied Maths, Sint-Martems-Latem, Belgium). The BioNumerics analysis was performed using the Dice coefficient and the unweighted pair group method of averages (UPGMA) with a 1% tolerance limit and 1% optimization. Isolates that clustered with ≥80% similarity were considered to belong to the same ERIC type, respectively.

### Statistical analysis

Data were transferred to a Microsoft Excel spreadsheet (version 15; Microsoft Corp., Redmond, WA, USA) for analysis. Using statistical software (version 16; SPSS Inc., Chicago, USA), Chi-square test and Fisher’s exact two-tailed test analysis was performed and differences were considered significant at values of *p* < 0.05.

## Results

Of 22 tested *A. baumannii* strains, 10 strains were originated from the chicken meat, 4 strains from bovine meat, 3 strains from camel meat, 3 strains from turkey meat and 2 strains from ovine meat.

**Fig. 1:**
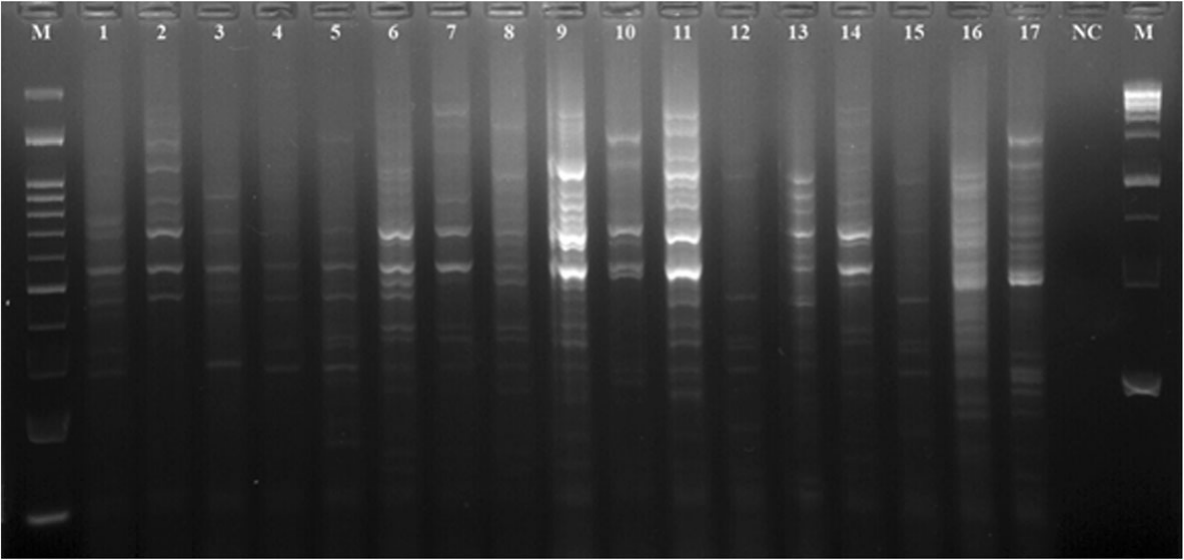
Electrophoretic patterns of the ERIC types of *A. baumannii* strains No 1–17 isolated from raw meat samples, NC=Negative control, M = DNA Ladder 100 bp, 1Kb

**Fig. 2:**
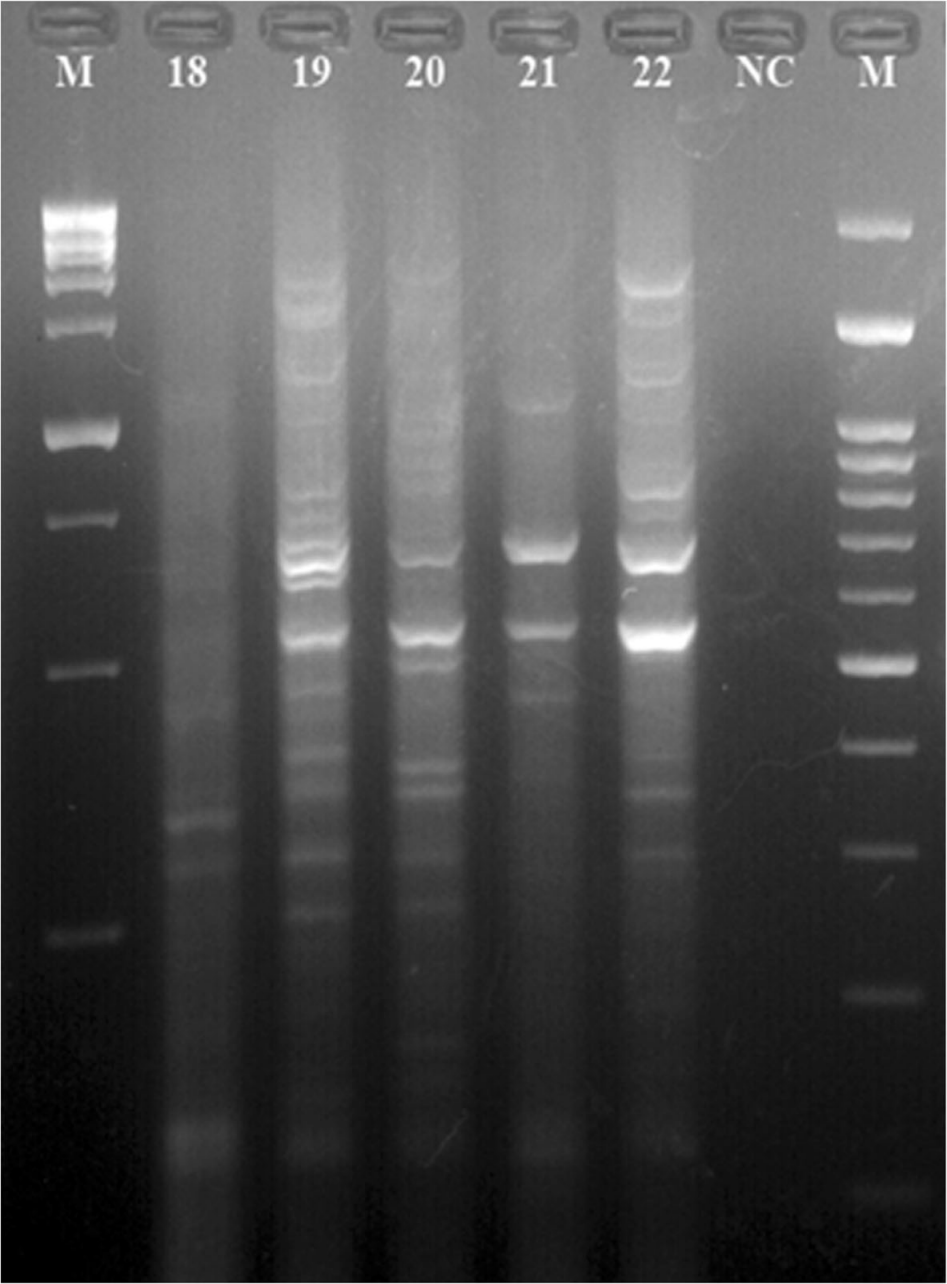
Electrophoretic patterns of the ERIC types of *A. baumannii* strains No 18–22 isolated from raw meat samples, NC=Negative control, M = DNA Ladder 100 bp, 1Kb

**Fig. 3:**
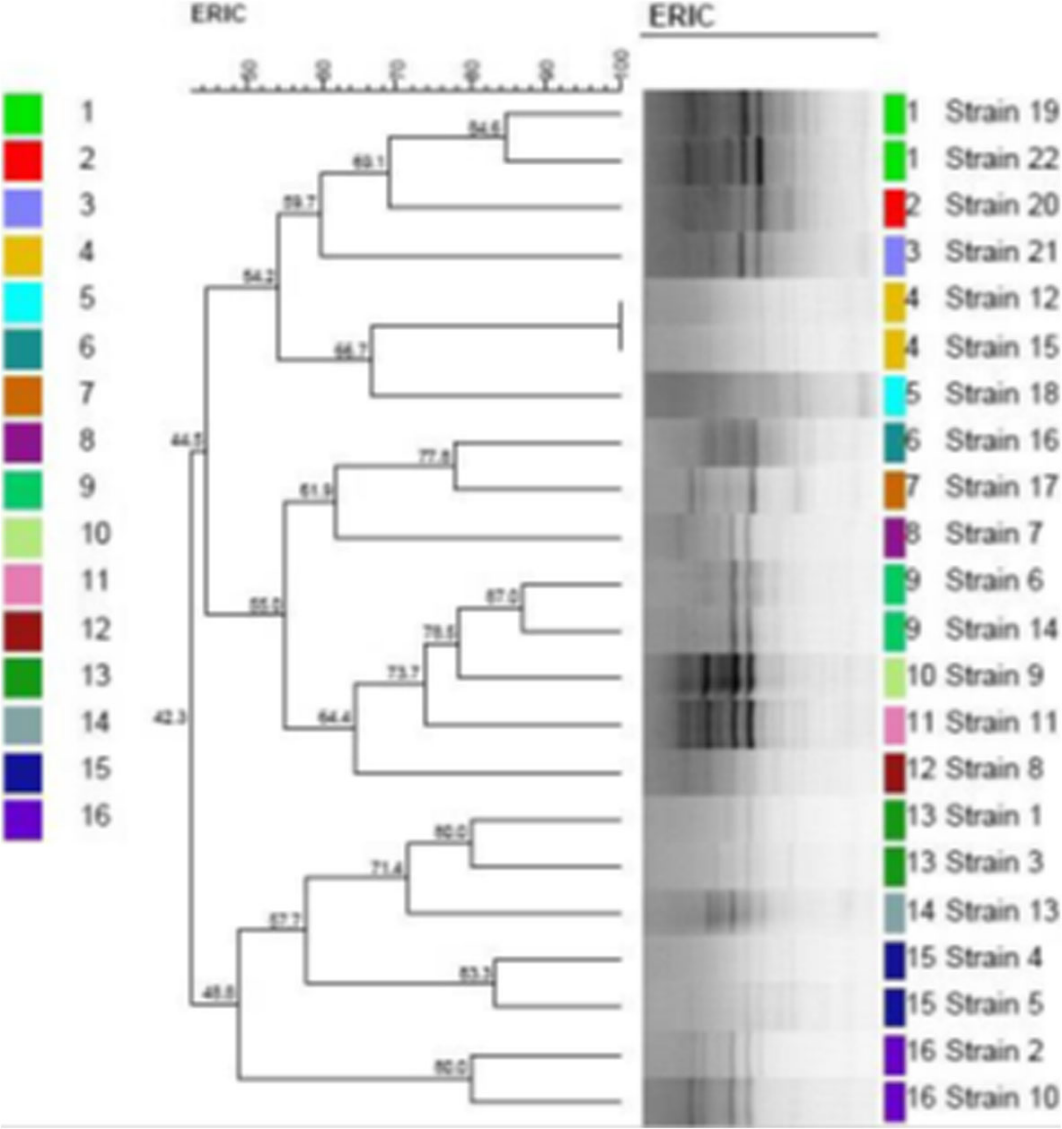
Dendrogram of genetic relatedness of the *A. baumannii* strains isolated from meat of animal origins using the ERIC-PCR

Figure 1 represent the electrophoretic patterns of the ERIC types of *A. baumannii* strains No 1-17 isolated from raw meat samples. Figure 2 represents the electrophoretic patterns of the ERIC types of *A. baumannii* strains No 18-22 isolated from raw meat samples. Figure 3 shows the results of the molecular typing of the *A. baumannii* strains isolated from different animal origins. *A. baumannii* strains with 80% and more than 80% similarity in the molecular typing were considered as one cluster. In keeping with this, a total of 16 ERIC types were identified. We found that strains no 19 and 22 (originated from chicken meat), 12 and 15 (originated from chicken meat), 6 (originated from bovine meat) and 14 (originated from chicken meat), 1 and 3 (originated from chicken meat), 4 (originated from bovine meat) and 5 (originated from turkey meat) and finally 2 (originated from chicken meat) and 10 (originated from ovine meat) had similar genetic cluster and were related to the 1, 4, 9, 13, 15 and 16 genetic cluster of the *A. baumannii* strains, respectively.

Table 2 represents the antibiotic resistance pattern of the *A. baumannii* strains isolated from different animal origins. *A. baumannii* strains harbored the highest prevalence of resistance against tetracycline (90.90%), trimethoprim (59.09%), co-trimoxazole (54.54%) and gentamicin (50.00%) antibiotics. *A. baumannii* strains of similar molecular cluster (ERIC-type) showed the same pattern of antibiotic resistance (Table 2)***.*** *TetA* (81.81%), *tetB* (72.72%), *dfrA1* (63.63%), *aac(3)-IV* (63.63%), *sul1* (63.63%) and *aadA1* (45.45%) were the most commonly detected antibiotic resistance genes. *A. baumannii* strains of the same molecular cluster had the same antibiotic resistance genes (Table 2).

**Table 2:** Profiles obtained by ERIC-PCR, virulence factors and antimicrobial resistance pattern in *A. baumannii* strains isolated from animal origins

ERIC types^a^	Numbers and source of strains	Antibiotic resistance phenotypes	Antibiotic resistance genes	®
I	19,22 (Chicken meat)	SXT25, TE30, F300	*aadA1, sul1, tetB, sim, oxa-58-like*	*cnf1, csgA, fimH*
II	20 (Chicken meat)	GM10, TE30, TMP5, CIP5, C30, F300	*sul1, tetA, tetB, dfrA1, qnr, cat1, vim*	*ibeA, sfa/focDE, afa/draBC*
III	21 (Chicken meat)	CL30, TE30, TMP5, E15	*tetB, dfrA1, qnr, cmlA,* *oxa-*23-*like*	*kpsMTII, PAI, ibeA, traT, afa/draBC, cnf2, csgA, fyuA, fimH*
IV	12,15 (Chicken meat)	S10, GM10, SXT25, TE30, TMP5	*aadA1, aac(3)-IV, sul1, blaSHV, tetA, dfrA1*	*kpsMTII, sfa/focDE, cnf1, iutA*
V	18 (Turkey meat)	TOB10, CL30, CAZ30, TE30, AZM15	*aac(3)-IV, blaSHV, ClTM, tetA, tetB*	*cvaC, fimH*
VI	16 (Ovine meat)	GM10, AK30, SXT25, CL30, CAZ30, TE30, TMP5, CIP5, LEV5, MEM10, C30, F300, E15	*aadA1, aac(3)-IV, sul1, blaSHV, ClTM, tetA, tetB, dfrA1, cat1, cmlA, sim, oxa-51-like, oxa-*23-*like*	*kpsMTII, ibeA, papC, papGII, papGIII, traT, afa/draBC, cnf1, cnf2, csgA, iutA, fimH*
VII	17 (Bovine meat)	S10, CAZ30, TE30, TMP5, IPM10, RA5	*aadA1, aac(3)-IV, ClTM, dfrA1, imp*	*PAI, papC, papGIII, traT, afa/draBC, cnf1, cnf2, csgA, cvaA, iutA, fyuA, fimH*
VIII	7 (Bovine meat)	F300, RA5	*aadA1, ClTM, tetA, tetB, dfrA1, cat1, imp,* *oxa-51-like, oxa-*24-*like*	*kpsMTII, PAI, papC, papGIII, traT, afa/draBC, cnf1, cnf2, csgA, iutA, fyuA, fimH*
IX	14 (Chicken meat) 6 (Bovine meat)	GM10, AK30, TOB10, CL30, TE30, CIP5, C30	*aac(3)-IV, sul1, blaSHV, tetA, tetB, dfrA1*	*papGII, papGIII, afa/draBC, cnf1, cnf2, cvaC, fyuA, fimH*
X	9 (Camel meat)	S10, AZM15	*aadA1, tetA*	*PAI, papC, papGII, sfa/focDE, afa/draBC, csgA, cvaC*
XI	11 (Camel meat)	GM10, TOB10, SXT25, TE30, C30, RA5	*aadA1, aac(3)-IV, sul1, tetA, tetB, cmlA*	*ibeA, papGII, sfa/focDE, traT, afa/draBC, cnf2, csgA, cvaC, fimH*
XII	8 (Chicken meat)	S10, AK30, CAZ30, TE30, LEV5, IPM10, C30	*sul1, ClTM, tetA, tetB, qnr, cat1, cmlA, vim, oxa-51-like, oxa-*23-*like,* *oxa-*24-*like*	*PAI, ibeA, papGII, sfa/focDE, cnf2, iutA, fimH*
XIII	1,3 (Chicken meat)	SXT25, TE30, TMP5, CIP5, MEM10, E15	*aac(3)-IV, sul1, ClTM, tetA, tetB, dfrA1*	*cnf2, csgA, iutA, fimH*
XIV	13 (Turkey meat)	TOB10, TE30, TMP5, CIP5, LEV5, E15	*aadA1, tetA, tetB, dfrA1, oxa-51-like,* *oxa-*24-*like*	*ibeA, papC, sfa/focDE, fimH*
XV	5 (Turkey meat) 4 (Bovine meat)	GM10, TOB10, SXT25, TE30, TEP5, E15	*aac(3)-IV, sul1, tetA, tetB*	*afa/draBC, cnf1, cnf2, csgA, cvaC, fimH*
XVI	2 (Camel meat) 10 (Ovine meat)	S10, GM10, TE30, TMP5	*aac(3)-IV, tetA, dfrA1,* *oxa-58-like*	*sfa/focDE, afa/draBC, cnf1, csgA, cvaC, iutA, fyuA, fimH*

^a^In this table: S10 = Streptomycin, GM10 = Gentamicin, AK30 = Amikacin, TOB10 = Tobramycin, SXT25 = Trimethoprim/sulphamethoxazole, CL30 = Cephalothin, CAZ30 = Ceftazidime, TE30 = Tetracycline, TMP5 = Trimethoprim, CIP5 = Ciprofloxacin, LEV5 = Levofloxacin, IPM10 = Imipenem, MEM10 = Meropenem, C30 = Chloramphenicol, F300 = Nitrofurantoin, AZM15 = Azithromycin, RA5 = Rifampin, E15 = Erythromycin

Table 2 also represents the distribution of virulence factors among the *A. baumannii* strains isolated from meat samples of animals. *FimH* (81.81%), *afa/draBC* (63.63%), *csgA* (63.63%), *cnf1* (59.09%), *cnf2* (54.54%) and *iutA* (50.00%) were the most commonly detected virulence factors amongst the *A. baumannii* strains isolated from animal origins. *A. baumannii* strains of same molecular cluster (ERIC-type) had the same virulence factors.

## Discussion

Acinetobacter is a nosocomial pathogen. Its ability to infect healthy hosts and its propensity to develop antimicrobial drug resistance is a cause for concern among infectious disease specialty. Although the ubiquitous existence of *A. baumannii* in hospital environment has been considered a routine delusion by several investigations [10, 12–14, 16, 17, 19, 21], different recent reports have undeniably highlighted the presence of the bacterium in different types of animal origins [11, 22, 23]. In recent years, nosocomial infections of *A. baumannii*, as an opportunistic pathogen, are increasing. Treatment of this bacteria especially multi-drug resistant and broad-spectrum beta-lactamases strains is of major concern [11, 22, 23]. These recent works have mainly been done only on the prevalence rate of *A. baumannii* and in some cases, on antibiotic susceptibility patterns. Reversely, to the best of our knowledge, the study presented here is the first report of the molecular typing, on the distribution of virulence factors and genotypic evaluation of antibiotic resistance of the *A. baumannii* strains isolated from different animal origins. A total of 22 strains of *A. baumannii* were assessed and 16 different genetic cluster were detected. Meat-derived *A. baumannii* strains may originate in the slaughterhouses, butchers and shopping centers especially due to the manipulation of the meat samples.

*A. baumannii* has previously been recognized as an animal colonizer with diverse distributions in different countries including in Scotland, 1.20% [22] and Senegal, 5.10% [24]. Rafei et al. (2015) [25] reported the high prevalence of *A. baumannii* strains in food samples with animal origins including raw meat, raw milk, and dairy products. The *A. baumannii* strains of the present research were frequently resistant to clinically relevant antibiotics. However, a few isolates displayed low levels of resistance against imipenem, azithromycin, meropenem, rifampin, levofloxacin, ceftazidime and tobramycin. This latter is still among the drugs of choice for the treatment of *A. baumannii* infections in humans and animals in Iran.

*A. baumannii* strains of the same molecular cluster (ERIC-type) had the same profile of the antibiotic resistance pattern*.* We also found that all of *A. baumannii* strains originated from chicken meat samples were susceptible to azithromycin and rifampin and resistant against tetracycline. These findings have been supported by several studies conducted on Lebanon [25], Switzerland [26], and France [27].

We found that molecular similarities of 19 and 22, 12 and 15, 6 and 14, 1 and 3, 4 and 5 and finally 2 and 10 *A. baumannii* strains were 84.60%, 100%, 87%, 80%, 83.30% and 80% with each other, respectively. All the other *A. baumannii* strains were classified as differing ERIC types. *A. baumannii* strains recovered from the bovine, camel, turkey and ovine meat samples didn’t show any similarity in their molecular typing and there were no similar antibiotic resistance patterns between them. Our findings showed that they also had similar phenotypic pattern of antibiotic resistance. One possible explanation for the similar molecular types of the *A. baumannii* strains recovered from different animal origins is their common source of infection. It may also be due to the close contact between bovine and chicken, bovine and turkey and between chicken and ovine species, facilitating transmission of the *A. baumannii* strains with same molecular type. Unfortunately, Iranian ranchers frequently maintain and breed different species of animals and especially bovine, ovine and caprine species in contact to each other. This may cause easy transmission of the *A. baumannii* strains between different species. The high genetic diversity of strains isolated from meat samples has also previously been reported by Lupo et al. (2014) [26] and Carvalheira et al. (2017) [28]. Recent research [21] revealed the similar antibiotic resistance pattern of the *A. baumannii* strains isolated from different types of raw meat samples. They showed that the prevalence of resistance against trimethoprim-sulfamethoxazole, tetracycline, amikacin, tobramycin, ampicillin-sulbactam, meropenem and imipenem were 23.20%, 23.20%, 14.30%, 12.50%, 12.50%, 8.30% and1.20%, respectively which was different to our findings. The prevalence of antibiotic resistant bacteria in meat samples has been attributed, at least partially, to the extensive use of antimicrobials for treatment, prevention and control of diseases and finally for growth stimulate in food-producing animals, since this enhances the antimicrobial selective pressure for strains present***.*** Otherwise, using antibiotics for growth stimulation is allowed in Iran. Low prevalence rate of resistance against carbapenems is due to the fact that these antibiotics are not allowed to treat food-producing animals.

Phenotypic characterization of antibiotic resistance was also confirmed by genotyping analysis of antibiotic resistance. We identified the genes for resistances to aminoglycosides (*aadA1* and *aac(3)-IV*), beta-lactams (*bla*_*SHV*_ and *bla*_*CTM*_), chloramphenicol (*cat1* and *cmlA*), tetracyclines (*tetA* and *tetB*), sulfonamides (*sul1* and *dfrA1*), carbenicilins (*imp*, *sim* and *vim*) and to fluoroquinolones (*qnr*). Furthermore, *A. baumannii* strains harbored their own specific antibiotic resistance genes (Table 2). This part of our study was in agreement with previous researches [21, 29]. Additionally, we found that *A. baumannii* strains of the same molecular cluster (ERIC-type) had the same profile of the antibiotic resistance genes.*,* as follows: strains no 1 and 3 of the same ERIC-type (type XIII) were positive for *CITM*,*dfrA1*, *tetA*, *tetB*, *aac(3)-IV* and *sul1* which showed their same genetic pattern of antibiotic resistance. Strains no 12 and 15 of the same ERIC-type (type IV) were positive for *tetA, aadA1, aac(3)-IV, blaSHV, dfrA1* and *sul1*. Strains no 19 and 22 of the same ERIC-type (type I) were positive for *tetB, aadA1, sim, oxa-58-like* and *sul1*. Despite of the different animal origin of the *A. baumannii* strains no 6 and 14 of the same ERIC-type (type IX), were positive for *tetA*, *tetB*, *aac(3)-IV*, *sul1, dfrA1* and *blaSHV* antibiotic resistance genes. Additionally, *A. baumannii* strains no 4 and 5 of the same ERIC-type (type XV) had different origins but they harbored *tetA*, *tetB, sul1* and *aac(3)-IV* antibiotic resistance genes. Finally, *A. baumannii* strains no 2 and 10 of the same ERIC-type (type XVI) were positive for *tetA, aac(3)-IV, dfrA1* and *oxa-58-like* antibiotic resistance. *A. baumannii* strains of other ERIC types had unique pattern of antibiotic resistance.

Virulence of *A. baumannii* is dependent on several other attributes (i.e. “slime”, LPS production and etc.) beside the selected virulence genes listed here [30, 31].

Another notable finding is the high prevalence of certain virulence factors in the *A. baumannii* strains isolated from meat of different animals. The most commonly detected virulence genes amongst these *A. baumannii* strains were *fimH*, *afa/draBC*, *csgA*, *cnf1*, *cnf2* and *iutA*. Prevalence of non-adhesive virulence factors including *traT* (serum resistance), *cvaC* (colicin V), *ibeA* (invasion), *fyuA* (yersiniabactin) and *PAI* (indicator gene) were low. This finding is in agreement with those of previous researches [30, 31]. Non-adhesive virulence factors play an important role in bacterial survival in special conditions such as in human blood and exposure to serum and poor iron environments contributing to the pathogenesis of extra instestinal diseases. However, presence of these non-adhesive virulence factors may not be essential for pathogenicity of food-borne enteric diseases. The gene *iutA* (aerobactin) has a high prevalence (50.00%) in our strains. Darvishi (2016) [32] reported that the prevalence of *cnf1*, *csgA*, *cvaC* and *iutA* virulence factors amongst the *A. baumannii* strains isolated from hospitalized patients were 50%, 70%, 10% and 25%, respectively. Daryanavard and Safaei (2015) [33] reported that the total prevalence of *csga*, *cnf1*, *cvaC* and *iutA* virulence genes among the samples of UTIs were 55%, 40%, 10% and 30%, respectively which was similar to our findings. Momtaz et al. (2015) [13] reported that the prevalence of *csga*, *cnf1*, *cvaC* and *iutA* virulence genes among the *A. baumannii* strains of clinical infections in Iran were 12.39%, 35.53%, 21.48% and 19%, respectively which was lower than our results.

We also found that the *A. baumannii* strains of the same molecular cluster (ERIC-type) had the same virulence factors. Despite of the different origin of the *A. baumannii* strains no 6 and 14 of the same ERIC-type (type IX), were both positive for *fimH*, *fyuA*, *cvaC*, *cnf1, cnf2*, *papGII, papGIII,* and *afa/draBC* virulence factors. Furthermore, *A. baumannii* strains no 4 and 5 of the same ERIC-type (molecular type XV) had different origins but they both harbored *fimH*, *cvaC*, *csgA, cnf1, afa/draBC* and *cnf2* virulence factors. Finally, *A. baumannii* strains no 2 and 10 of the same ERIC-type (type XVI) were positive for *fimH*, *fyuA*, *csgA*, *afa/draBC*, *cvaC, iutA, cnf1* and *sfa/focDE* virulence factors. These high similarities in the pattern of virulence factors of the *A. baumannii* strains of same ERIC types recovered from different origins showed that genetic cluster of bacterial strains is closely related to their virulence determinants.

*A. baumannii* strains isolated from the chicken meat samples had similar molecular type. *A. baumannii* strains isolated from other meat samples did not fall into common molecular types. Two *A. baumannii* strains of bovine origins had similar molecular type with turkey and chicken. A camel and ovine *A. baumannii* strains had also similar molecular type.

## Conclusions

In conclusion, we studied the phenotypic and genotypic patterns of antibiotic resistance of the *A. baumannii* strains isolated from meat samples of different animals. High prevalence of resistance against tetracycline, trimethoprim, co-trimoxazole and gentamicin antibiotics and high distribution of *tetA*, *tetB*, *dfrA1*, *aac(3)-IV*, *sul1* and *aadA1* antibiotic resistance genes was established. Besides, *fimH*, *afa/draBC*, *csgA*, *cnf1*, *cnf2* and *iutA* were the most commonly detected virulence factors amongst the *A. baumannii* strains of our study. Twenty-two *A. baumannii* strains were classified into the 16 different genetic clusters according to the results of the ERIC-PCR. We also found that genetic cluster of the strains was closely related to their antibiotic resistance genes and virulence factors. Similarities in the genetic cluster of some strains was also recorded which may represent their similar sources of contamination.

## Abbreviations

CLSI: Clinical and Laboratory Standards Institute
ERIC-PCR: Enterobacterial Repetitive Intergenic Consensus-Polymerase Chain Reaction
